# An Improved Canine Genome and a Comprehensive Catalogue of Coding Genes and Non-Coding Transcripts

**DOI:** 10.1371/journal.pone.0091172

**Published:** 2014-03-13

**Authors:** Marc P. Hoeppner, Andrew Lundquist, Mono Pirun, Jennifer R. S. Meadows, Neda Zamani, Jeremy Johnson, Görel Sundström, April Cook, Michael G. FitzGerald, Ross Swofford, Evan Mauceli, Behrooz Torabi Moghadam, Anna Greka, Jessica Alföldi, Amr Abouelleil, Lynne Aftuck, Daniel Bessette, Aaron Berlin, Adam Brown, Gary Gearin, Annie Lui, J. Pendexter Macdonald, Margaret Priest, Terrance Shea, Jason Turner-Maier, Andrew Zimmer, Eric S. Lander, Federica di Palma, Kerstin Lindblad-Toh, Manfred G. Grabherr

**Affiliations:** 1 Science for Life Laboratories, Department of Medical Biochemistry and Microbiology, Uppsala University, Uppsala, Sweden; 2 Broad Institute of MIT and Harvard, Cambridge, Massachusetts, United States of America; 3 Division of Nephrology, Massachusetts General Hospital and Harvard Medical School, Charlestown, Massachusetts, United States of America; 4 Memorial Sloan-Kettering Cancer Center, New York, New York, United States of America; 5 Boston Children's Hospital, Boston, Massachusetts, United States of America; 6 Department of Cell and Molecular Biology, Uppsala University, Uppsala, Sweden; 7 Vertebrate and Health Genomics, The Genome Analysis Centre, Norwich, United Kingdom; Florida State University, United States of America

## Abstract

The domestic dog, *Canis familiaris*, is a well-established model system for mapping trait and disease loci. While the original draft sequence was of good quality, gaps were abundant particularly in promoter regions of the genome, negatively impacting the annotation and study of candidate genes. Here, we present an improved genome build, canFam3.1, which includes 85 MB of novel sequence and now covers 99.8% of the euchromatic portion of the genome. We also present multiple RNA-Sequencing data sets from 10 different canine tissues to catalog ∼175,000 expressed loci. While about 90% of the coding genes previously annotated by EnsEMBL have measurable expression in at least one sample, the number of transcript isoforms detected by our data expands the EnsEMBL annotations by a factor of four. Syntenic comparison with the human genome revealed an additional ∼3,000 loci that are characterized as protein coding in human and were also expressed in the dog, suggesting that those were previously not annotated in the EnsEMBL canine gene set. In addition to ∼20,700 high-confidence protein coding loci, we found ∼4,600 antisense transcripts overlapping exons of protein coding genes, ∼7,200 intergenic multi-exon transcripts without coding potential, likely candidates for long intergenic non-coding RNAs (lincRNAs) and ∼11,000 transcripts were reported by two different library construction methods but did not fit any of the above categories. Of the lincRNAs, about 6,000 have no annotated orthologs in human or mouse. Functional analysis of two novel transcripts with shRNA in a mouse kidney cell line altered cell morphology and motility. All in all, we provide a much-improved annotation of the canine genome and suggest regulatory functions for several of the novel non-coding transcripts.

## Introduction

The dog, *Canis familiaris*, is an important and well-established genetic model used to study human disease. Since dogs were first domesticated more than 10,000 years ago [1]–[4], they have shared their immediate surroundings with humans, and as a consequence have been exposed to many of the same pathological, dietary [1] and environmental conditions. In only a few centuries, hundreds of dog breeds have been created and continuously selected to generate diverse morphological, physiological and behavioral variation. However, an unintended side effect to this process has also led to the accumulation of certain health disorders within specific breeds. In 2005, the first high-quality draft genome was published accompanied by a SNP discovery effort and the characterization of haplotype structure, as well as power calculations for strategies for genome-wide association mapping of trait and disease loci [5]. Intense breeding has resulted in short linkage disequilibrium (LD) across breeds but long LD within breeds, thus making the pure bred dog an ideal model to study disorders that also affect humans through genome wide association [5], [6].

Multiple SNP arrays were designed based on the canine draft genome assembly and these were effectively used to map by genome-wide association (GWAS) some of the phenotypically plastic traits under selection, including coat color [7], [8] and morphology [9]–[11]. Whilst those studies offered insight into basic biology (pigmentation, bone formation), parallel investigations into specific heritable diseases suffered by humans and their canine companions revealed novel pathways (NFAT in SLE-like disease [12], *HAS2* biosynthesis in autoinflammatory disease [13]) and candidate genes for disease biology such as *LGI2* in remitting focal epilepsy [14], and *SLC4A3* in progressive retinal atrophy [15]. The mutations identified to date include both changes in proteins, as well as alterations in gene expression regulation.

While GWAS is relatively effective in dogs, the identification of actual mutations has been more difficult for several reasons. First, there are many gaps in the draft genome caused by sequencing bias against GC-rich regions, particularly in promoters and first exons. It has been proposed that the high GC content may have been caused by the loss of *PRDM9* in the canine lineage, in turn leading to sustained recombination hotspots and GC biased gene conversion in these regions [16]. Secondly, a comprehensive catalog of genes, non-coding transcripts, and regulatory elements is currently not available, a resource that would be greatly beneficial when identifying the causal mutations of complex diseases, many of which are likely regulatory in nature. For example, expression levels of immunoglobulin E antibodies have been linked to allergic reactions, and while two GWAS signals have been found [17], no obvious gene candidates were annotated in these regions. This, among other examples, highlights how the potential of mutation identification is limited by the quality of the genome, as well as the set of transcribed and characterized features.

Here, we sought to improve the assembly quality and completeness, as well as transcript annotation for both coding and non-coding genes. We first present canFam3.1, a new genome build in which thousands of gaps were filled. In addition, we present a new annotation, which greatly expands the repertoire of known dog transcripts, including protein coding genes, putative long intergenic non-coding RNAs (lincRNAs), and antisense transcripts. This resource will now be available to the genetics community (http://genome.ucsc.edu/cgi-bin/hgHubConnect “Broad Improved Canine Annotation v1”).

## Results

### Improved quality and contiguity of the canine genome

Despite the overall high quality of the dog canFam2.0 assembly, ∼1% of the euchromatic genome resides in gaps and ∼0.5% of the assembly shows evidence of potential assembly errors. In addition, ∼20% of genes show some evidence of sequencing errors, such as frameshifts, very short introns, and missing first exons and promoters. To improve the quality and completeness of the canine genome sequence, we targeted the following regions: (a) small assembly gaps, many of which are caused by either simple sequence repeats, or cloning bias against high GC content; and (b) long-range assembly gaps or errors that are likely part of any draft genome produced from whole-genome shotgun sequencing [18], predominantly caused by transposable elements and segmental duplications. For closing small gaps, we employed 12,101 primer walks (see Methods), while we used re-sequencing of Bacterial Artificial Chromosomes (BACs) to close large gaps and cover mis-assembled regions. For BAC re-sequencing, we selected (a) 283 gaps larger than 35 kb; (b) 100 clusters of gaps less than the BAC size of 180 kb; (c) 22 gaps between adjacent scaffolds anchored to the same chromosome; (d) 59 centromeric and telomeric regions; and (e) 65 regions indicated as being problematic by the whole genome shotgun assembler Arachne [19] which were of ≥50 kb, or which fell close to a gap of size 10–35 kb. This totaled 149 BACs. In addition, we picked 186 BACs to cover the dog homologs to the 45 human ENCODE pilot regions [20]. In total we integrated 85 Mb (an ∼0.4% increase) of novel sequence from BAC and primer walks into the canFam2.0 assembly to create canFam3.1 (see Methods, integration of improved sequences). The new assembly version has 14,418 gaps, 95% of which are less than 2 kb, while the amount of sequence currently flagged as potentially mis-assembled is less than 0.2% (4.1 Mb) of the assembly (Table 1). The new genome build closed a total of 1,044 gaps containing promoters and/or first exons of protein coding genes that were missing in canFam2.0.

**Table 1:** Comparison of canFam2.0 and canFam3.1.

Property	canFam2.0	canFam3.1
Coverage of euchromatic portion of genome (%)	99.2	99.6
Portion of assembly in “certified regions” (%)	99.5	99.8
Contiguity: gaps per Mb	12	6
ENCODE regions	High-quality draft	98% Finished

### Generating a transcriptome

We performed deep RNA sequencing (RNA-Seq) to generate a comprehensive catalogue of transcripts for the dog genome, to both augment the set of known protein coding genes, as well as to denote and characterize non-coding transcripts that have previously not been listed in the EnsEMBL annotation. We used ten different dog tissues: blood, brain, heart, kidney, liver, lung, ovary, skeletal muscle, skin, and testis (Table S1) to create two sets of libraries: (a) strand-specific dUTP with poly-A selection [21], which specifically captures protein coding genes and other transcripts transcribed by polymerase II by targeting the poly-adenylated transcript tails; and (b) duplex-specific nuclease (DSN), which substantially reduces the levels of the highly abundant ribosomal transcripts through normalization, and does not depend on the mechanism of transcription [22]. We sequenced each sample using the Illumina HiSeq platform, generating between 30 and 100 million paired-end reads of 101 bp each. We mapped all reads from the resulting 21 libraries (brain and kidney were generated in duplicates for poly-A, and the DSN skin library was excluded due to poor alignment performance) to the canFam3.1 genome build using the splice junction mapper Tophat (version 2.0.5) [23] (Table S1), and assembled transcript models using the Cufflinks package (version 2.0.2) [24] without guidance from any existing annotation.

Overall, we identified 65,314 loci from all poly-A selected samples combined, with testis showing the highest number of expressed loci (41,070 loci, Table 2). This agrees with previous observations showing that polymerase II activity is elevated in the germ-line [25]. The overlap of expressed loci between any two tissues sequenced from the poly-A libraries varied from 50% to 90% depending on the pair-wise tissue comparison (Table S2-A). The mean transcript size was 3169 bp (median 2173 bp). Related tissues such as skeletal muscle and heart shared the largest number of loci (88.4%). In contrast, the DSN libraries revealed a substantially larger number of total loci (194,878) ranging from 33,857 in testis to 96,231 in liver (Table S2-B), but the transcripts had a smaller mean transcript size of 1485 bp (median 809 bp). The combined set of poly-A and DSN data resulted in 249,889 transcripts contained in 174,336 loci. Of these, 99,468 (57%) loci containing 150,034 transcripts showed a measurable (FPKM>0.1) expression in both library preparation types.

**Table 2:** Transcribed loci per tissue and library preparation.

	Brain	Blood	Heart	Kidney	Liver	Lung	Muscle	Ovary	Skin	Testis	Total
**Poly-A**	30,325	33,486	23,807	28,420	25,431	29,493	39,976	22,221	34,335	41,070	65,314
**DSN**	69,030	64,657	43,659	38,059	96,231	67,842	31,006	83,665	N/A	33,857	194,878

N/A, due to poor alignment performance, this library was excluded from subsequent analyses.

In the following sections, we categorize the transcripts, both through comparison with known features in dog and human, as well as by their genomic locations, splicing, and expression patterns.

### An extended catalog of protein coding genes

The products of protein-coding genes, which are transcribed by polymerase II, constitute the most well-characterized fundamental building blocks of life. Out of the 19,856 loci that are annotated by EnsEMBL (build 64) as such in the canine genome, 17,715 (89%) showed measurable expression (FPKM>0.1, i.e. >1-fold physical read coverage) in at least one sample and one library construction method in our data set. About 76% of loci were reported by both poly-A and DSN, 20% by poly-A only, while only 3.1% were detected by DSN but not poly-A. To recover protein-coding genes missed by the dog EnsEMBL annotation, we syntenically mapped all protein coding genes from the human genome (Hg19, EnsEMBL 68) to canFam3.1 using the synteny aligner *Satsuma* [26] and the coordinate mapper *RUM* (Zamani et al., submitted). We found an additional 2,942 loci supported by our RNA-Seq data in which protein coding annotations existed in the human orthologous regions, bringing the total number of ‘high-confidence protein coding genes’ to 20,657, which is comparable to the 21,329 annotated protein coding genes in the human genome (Table S3). Moreover, RNA-Seq estimated an average of 4.7 alternative isoforms per locus, which is similar to human and mouse annotations in EnsEMBL [27], and much higher than the 1.27 isoforms per dog gene as annotated by EnsEMBL. However, the number is still smaller than the 10–12 isoforms per cell line that have been observed [28] through much deeper sequencing of RNA. A total of 2,141 EnsEMBL protein-coding genes (2,741 transcripts) did not have any measurable support in the RNA-Seq data. Since it is possible that at least a fraction of these genes are correct predictions, but were not detectable in the data, as they could be expressed in a very tissue-specific manner (e.g. olfactory receptors), or only during early developmental stages, we included these loci in the list of protein coding genes, totaling 22,798 loci.

### A wealth of antisense transcripts

Antisense transcription has been reported to facilitate a more fine-tuned regulation of gene expression, usually of the protein coding gene located sense to the antisense element [29], [30]. In our RNA-Seq data set, we found 4,636 sequences that were transcribed from the opposite strand of a protein-coding gene and which also overlapped with at least one sense exon. More than half of the transcripts (2,379, 51.3%) featured multiple exons. Antisense transcripts showed more tissue-specific patterns compared to protein coding genes, with only 15 antisense loci being expressed in all samples and tissues (FPKM>0.1) and 994 (21.4%) specific to a single tissue. To determine whether antisense transcripts are associated with tissue-specific cellular processes, we analyzed their protein coding host genes using the Ingenuity Pathway Analysis package (Ingenuity Systems, http://www.ingenuity.com). The analysis revealed a diverse range of gene functions and many generic networks, such as ‘DNA replication and repair’ (39 molecules), ‘post-translational modification’ (37 molecules), and ‘nervous system development’ (37 molecules) (Table S4). The most prominent canonical pathway is ‘protein kinase A signaling’ (p<2.7×10^−4^) and ‘other cell-to-cell communication pathways’ (p<3×10^−3^, Table S5). These findings would suggest that the cellular processes subject to antisense regulation were ubiquitous and not associated with networks particular to any tissue type, but rather that the tissue-specific antisense expression patterns could be utilized to fine-tune these basic processes in different cell types and cell states.

### Long intergenic non-coding transcripts

**Figure 1:**
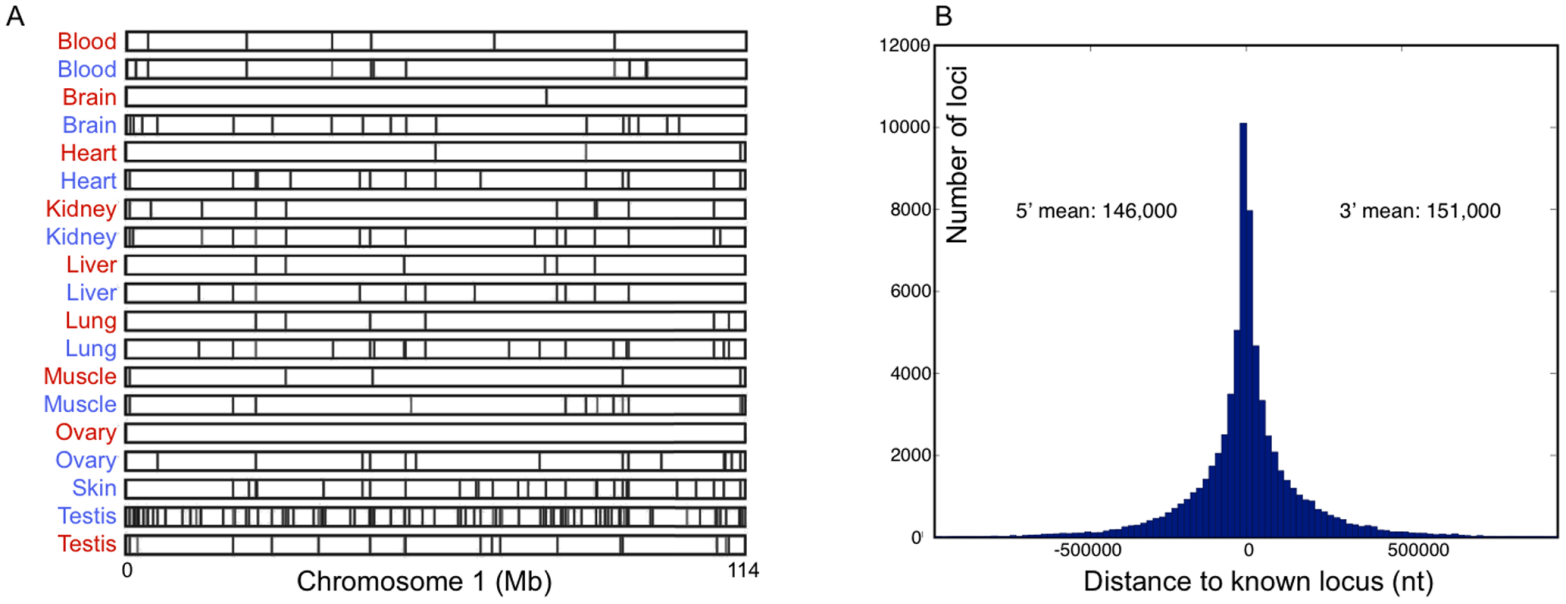
Location of lincRNAs and single-exon intergenic non-coding transcripts. (a) We show the mapping of lincRNAs, broken down by sample across chromosome 1. (b) Histogram of distance from intergenic transcripts to the next known transcribed element in both the 5′ (left) and 3′ (right) direction. The average distance is around 150,000 nucleotides, suggesting that these loci are not closely associated with known genes.

Thousands of long intergenic non-coding transcripts (lincRNAs) have been identified in the human and mouse genomes in the past few years, but the function has only been defined for a small subset, which are involved in the modulation of gene expression [31], imprinting [32], [33], and embryonic development [34]. Here we define canine lincRNAs as transcripts that (a) have no recognizable protein coding potential (see Methods); (b) do not overlap with either high- or low-confidence protein coding genes; and (c) have a spliced multi-exon structure. We found a total of 7,224 intergenic transcripts that fit these criteria (between 2 and 15 exons), and of these, 5,916 had not previously been annotated in either dog or human (see Methods). The mean transcript size was 1,894 bp (median 1,129 bp) and the mean span on the genome 19.3 kb (median 7.7 kb). Similar to protein coding genes, their distribution is uneven across the genome and differs between tissues, as exemplified by their locations on dog chromosome 1 (Figure 1A).

In line with previous findings [35], lincRNAs appeared to be highly tissue specific, with 37% of transcripts showing single tissue expression (tau = 1) [36]). The average tau score was 0.96 for lincRNAs as compared to 0.82 for protein-coding genes and 0.90 for antisense transcripts. Neighboring protein-coding genes are enriched for canonical pathways involved in development and differentiation (Wnt/β-catenin Signaling 3.3×10^−6^, Factors Promoting Cardiogenesis in Vertebrates 1.3×10^−4^, Mouse Embryonic Stem Cell Pluripotency 2.7×10^−4^, Human Embryonic Stem Cell Pluripotency 4.5×10^−5^) agreeing with previous studies [35], [37], but also for genes regulating the immune-system (Role of Macrophages, Fibroblasts and Endothelial Cells in Rheumatoid Arthritis 2.5×10^−5^, see Table S6).

### Other non-coding transcripts

In contrast to protein coding genes, antisense transcripts and long intergenic non-coding RNAs, there is little systematic knowledge about transcribed loci that do not fit into any of these categories. The dog EnsEMBL annotation lists 4,724 such loci as non-coding, with an additional 12,736 loci having orthologs annotated as microRNAs, snoRNAs, pseudogenes, etc. in the human EnsEMBL annotation. Out of those, only 1,818 (931 dog, 887 human orthologs) showed expression at FPKM>0.1 in the RNA-Seq data set generated here. By contrast, we found 137,996 transcripts expressed above that threshold that have not previously been described before in the dog or human genome. In terms of genomic location, the largest category is comprised of intronic transcripts on the same strand as their spliced coding (92,657 loci, 67.1%) and non-coding (3,735 loci, 2.7%) host genes, suggesting that these transcripts are artifacts of incompletely processed introns. We note that 65% of these loci were reported by both library construction methods, 33% by DSN only, and that the poly-A fraction is an almost perfect subset of the DSN sequences with only 1.8% of loci being detected by poly-A but not DSN. This finding would be consistent with the poly-A protocol also targeting incompletely spliced intronic sequences that are rich in the nucleotide adenosine. An additional 6,908 transcripts are located in introns of protein coding genes but in the antisense direction. Unlike intronic sense transcripts, only 0.05% of which are spliced, ∼30% of intronic antisense sequences feature multiple exons, suggesting that these transcripts might be unconnected fragments of antisense transcripts as described above or potentially uncharacterized unique transcripts.

Of the remaining 41,584 single-exon transcripts located in intergenic regions that were reported by the RNA-Seq data but not supported by known features, we found a fraction that were located in close proximity to known genes, including (a) 9,775 loci located on the same strand as the nearest genes and within 25 kb downstream of its 3′ transcription stop site, noting that these could be the by-product of spurious transcription beyond the stop site (mean expression: FPKM 2.4); and (b) 2,961 loci on the reverse strand to the nearest protein coding genes and within 5 kb region upstream of the transcription start, perhaps the result of promoter bi-directional activity (mean expression: FPKM 2.3). However, the majority of intergenic transcripts (28,868, or 69.4%) are not strongly co-located with protein coding genes (mean distance 150 kb in both the 5′ and 3′ direction (Figure 1B)). This agrees with previous findings that in general, non-coding RNAs are not strongly co-located with protein coding genes [38], but also that these transcripts might be actively and independently transcribed, rather than being the byproduct of nearby protein coding gene transcription. However, we note that only 38% of these loci are reported by both library construction methods, with 32.1% detected by DSN only, and 26.4% by poly-A only, thus we cannot rule out that a fraction of these intergenic transcripts could be artifacts originating from genomic DNA.

### Distinct expression patterns for coding, antisense, lincRNA, and intergenic loci

**Figure 2:**
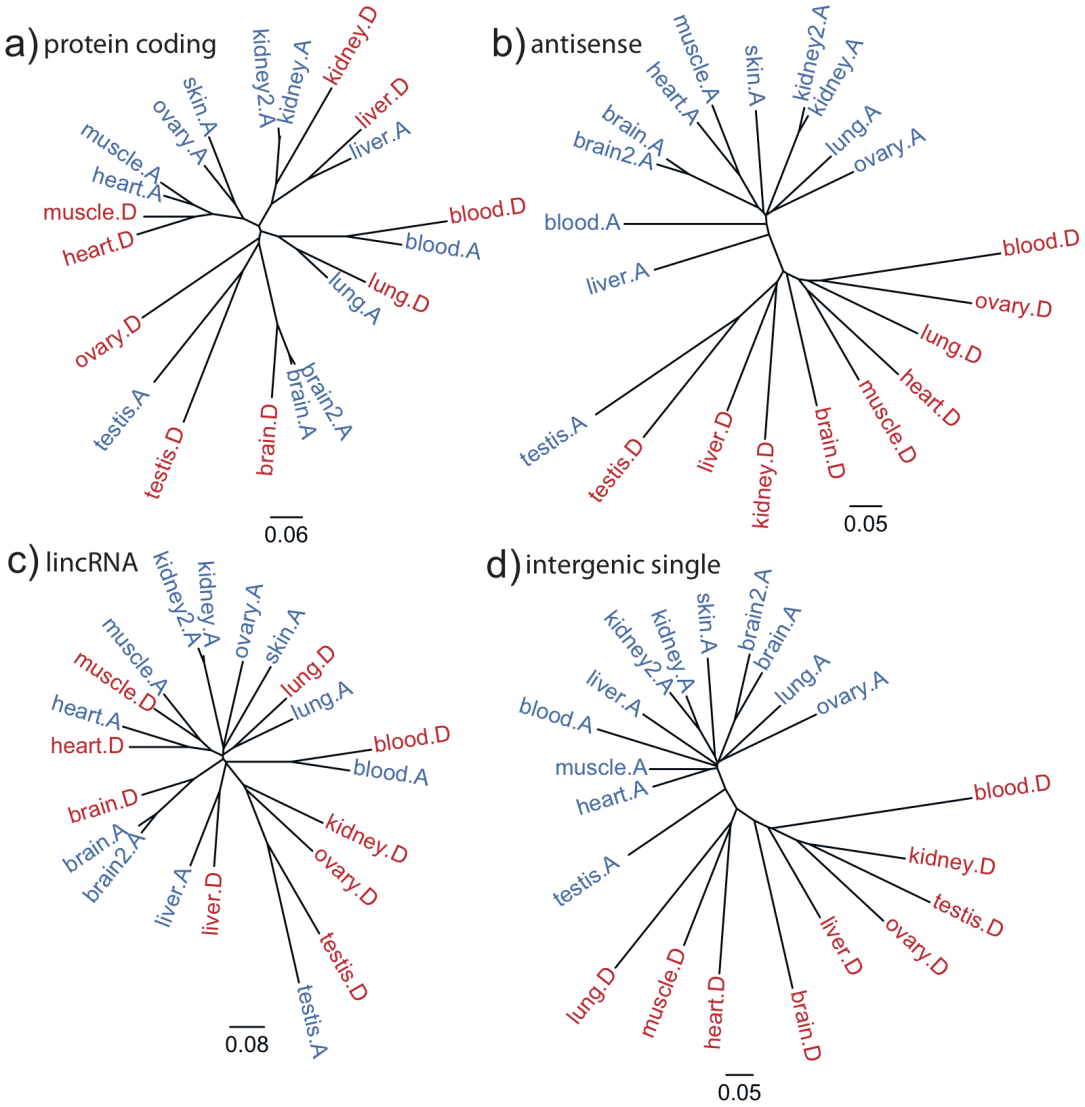
Distance trees of expression profiles. We constructed neighbor-joining trees based on the correlation between expression values (FPKM>1.0) between samples, with 1 minus Spearman's rho defining the distance. Colors denote library construction methods (poly-A: blue, DSN: red). We divided transcribed loci into (a) protein coding genes with RNA-Seq support, either annotated by EnsEMBL in dog or EnsEMBL in the human orthologous regions. Replicates cluster together, so do the library constructions methods poly-A and DSN, as well as related tissues, such as heart and muscle; (b) antisense transcripts, that overlap at least one exon of a protein coding gene, as defined in (a). With the exception of testis, poly-A and DSN separate the samples, with both the poly-A and DSN sub-trees maintaining closer relationships between the related tissues heart and muscle; (c) spliced intergenic loci, excluding sequences that have coding potential. Similar to protein coding genes, the poly-A and DSN group by tissue first, with the exception of kidney DSN; and (d) intergenic and uncharacterized single-exon transcript loci. In this set, DSN and poly-A are, similar to antisense loci, the most dominant factor when grouping samples.

An examination of gene expression patterns within and across tissues may indicate potential biological roles for different transcript types. Overall, protein coding genes are more highly expressed than non-coding transcripts: at the 95th percentile of expression, protein coding genes show an FPKM value of 48.0, while intergenic non-coding loci exhibit 10-fold lower levels (FPKM = 5.6), and antisense loci being transcribed at even lower levels (FPKM = 3.2) (Figure S1). Neighbor-joining (NJ) phylogenies (Figure 2) were drawn to investigate trends in expression patterns for each class of molecule and each library type (poly-A, blue; DSN, red). The distance between two samples was calculated as one minus Spearman's rank correlation coefficient (rho), based on FPKM values (methods). The NJ tree groups protein coding genes (Figure 2a) first by tissue, whereas a tree computed from antisense transcripts (Figure 2b) grouped samples by library construction first, with testis being the only exception. Expression of spliced lincRNAs (Figure 2c) resembles the pattern of protein-coding genes, indicating tissue specificity comparable to protein coding genes, even though lincRNAs are expressed at lower levels overall. By contrast, the unspliced ∼41,000 intergenic RNAs (Figure 2d) follow a pattern similar to antisense transcripts. Altogether this implicates intriguing differences in regulation of expression by protein-coding genes and lincRNAs versus antisense transcripts and single exon intergenic transcripts.

### Validation of non-coding RNA loci

In order to validate the novel non-coding RNA transcripts we selected 13 intergenic and 9 antisense loci, which were primarily expressed in one tissue at a FPKM>1, and performed quantitative RT-PCR in a panel of 10 tissues from up to 4 different dogs (24 samples total: brain, either cerebellum or cortex; gonad, ovary or testis; heart; kidney; liver; lung; pancreas; skin, see Methods). All 22 transcripts except one (96%) were detected in the discovery tissue, although seven transcripts (32%, primarily those discovered in brain and gonads) were not detected in all samples of that particular tissue, suggesting that expression may be regulated by region or cell state within the tissue (Tables S7, S8). Curiously, all transcripts also showed expression, often at low levels, in one or more other tissue, supporting the notion that these transcripts may be active in tissues other then the one in which they were discovered. This may also indicate a sample specificity stemming from temporal changes in expression, or the different conditions in which the samples were collected. Nonetheless, these data solidly support the expression of these transcripts, consistent with the potential for biological function.

### Cross-species functional analysis of novel non-coding kidney specific transcripts

The canine multi-tissue RNA-seq data presented here are consistent with results from other species, in that they suggest a multitude of non-coding RNA sequences to be actively transcribed [28]. To test whether orthologous sequences are also transcribed in other species, mouse and human, we selected nine multi-exonic canine transcripts, seven antisense and two lincRNAs, previously not seen in human or mouse. The transcripts were expressed in kidney in our RNA-Seq data and were therefore amplified in dog kidney as well as in mouse and human kidney RNA by RT-PCR, using primers from orthologous regions (see methods). Six of the seven antisense multi-exon canine loci expressed in canine kidney were amplified from human kidney RNA and both lincRNAs were also present in human and mouse kidney RNA (Table S9).

**Figure 3:**
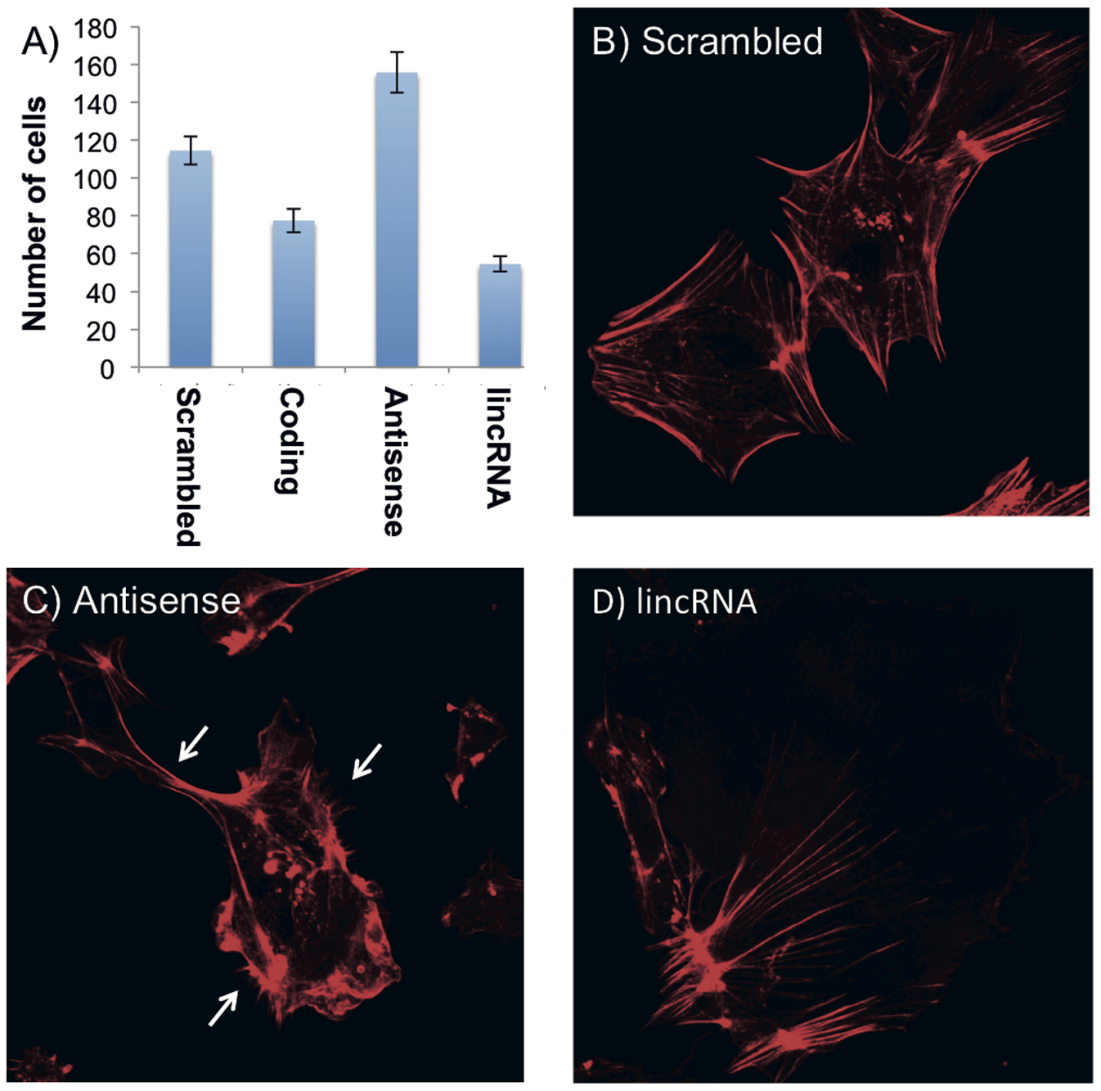
Modulation of podocyte motility through shRNAs to non-coding RNAs at the *BAIAP2* locus. A) The number of cells (average +/− SEM) that migrated into the scratch after 48 hours for each of the four conditions: scrambled shRNAs (negative control), shRNAs directed at coding sequence (positive control), antisense shRNAs, and lincRNA shRNAs. As previously shown, shRNA to *Baiap2* (coding shRNA) inhibited podocyte migration (p<0.01). shRNAs to the lincRNA further reduced podocyte migration (p<0.001 compared to scrambled shRNA, p<0.01 compared to coding shRNA), while shRNAs to the antisense transcript resulted in increased podocyte motility (p<0.01). (B–D) Phalloidin (actin) stained podocytes treated with B) scrambled shRNAs (normal podocyte appearance), C) antisense shRNAs and D) lincRNA shRNAs. Treatment with shRNAs to the antisense (C) resulted in increased filapodia formation (white arrows), consistent with increased *BAIAP2* activity. Treatment with lincRNA shRNAs (D) resulted in an abnormal appearance of the podocytes and the actin cytoskeleton.

To assess the potential function of a subset of novel transcripts, we chose two novel spliced non-coding RNAs (one antisense and one lincRNA) based on their location near *Baiap2*, a gene for which the effect of its expression level has previously been described in a murine podocyte cell model [39]. *Baiap2* is essential for filapodia formation, actin cytoskeleton formation/stability, and for the motility of kidney podocytes (part of the glomerulus in the kidney). It has been shown that the over-expression of *Baiap2* increased filapodia formation, while gene knockdown resulted in decreased murine podocyte migration as assessed by wound healing assay [39]. We designed multiple shRNAs for the antisense intronic transcript (located in intron 5), and the lincRNA located at the 5′ end of the gene, ∼10 kb away. Podocytes were infected with snRNA containing lentivirus and cell migration (wound healing) was measured at 48 hours. As expected [39], shRNAs targeting the coding region of *Baiap2* resulted in decreased migration. shRNAs directed against the lincRNA resulted in a further reduction in podocyte migration and alteration of the actin cytoskeleton (Figure 3), whilst increased filapodia formation and increased podocyte migration were observed with shRNAs directed against the intronic antisense transcript. Quantitative PCR confirmed reduced expression of *Baiap2* in podocytes treated with shRNAs to the coding region and the lincRNA, but unchanged expression in podocytes treated with shRNAs to the antisense transcript (Figure S2). Further studies are required to determine if these transcripts have *cis* effects at the *Baiap2* locus, or if they perform a more global modulation of podocyte gene expression.

## Discussion

The dog genome is important both for mammalian comparative sequence analyses and for use as a model for disease trait mapping [40], [41]. Identifying the underlying genomic causes for disease susceptibility at high resolution requires both a high quality genome sequence, as well as a comprehensive catalogue of transcribed features and regulatory elements. Here, we present an improved genome build, canFam3.1, together with a comprehensive annotation of protein coding and non-coding genes. Through deep sequencing of RNA obtained from ten different tissues, we significantly expanded the set of protein coding genes, both in terms of the number of loci and in alternative isoforms per gene, with the latter representing a four-fold increase of that previously listed by EnsEMBL.

It is well accepted that variants that alter amino acid sequences, introduce frame shifts and/or premature stop codons can deleteriously affect protein and cellular function. However, more and more data suggest that regulatory mutations are perhaps more important for understanding complex human diseases. It is therefore paramount to pinpoint mutations that affect the regulatory machinery, including regulatory transcripts of different types.

Antisense transcripts, which overlap with protein coding genes, frequently regulate their host genes, and we now annotate thousands of such loci in the dog genome for the first time. Likewise, transcribed and spliced, but non-protein-coding transcripts, termed lincRNAs, constitute a putative class of transcripts that have been linked to modulation of gene expression [31], imprinting [31]–[34], [42], or protein scaffolding [37], [43]. While none have previously been reported in the dog genome, we now list the locations of more than 7,000 lincRNAs, of which only a little over a thousand have previously annotated orthologous counterparts in the human genome. Interestingly, we found that, unlike antisense transcripts, the expression patterns of lincRNAs more closely resemble those of protein coding genes, indicating that the implied ‘regulatory roles’ of lincRNAs and antisense transcripts might be fundamentally different; this notion is further supported by GO term analysis of the host genes of antisense loci, which suggests that these transcripts are, at least in part, involved in the regulation of fundamental cellular processes common to cells of different tissue types, whereas the lincRNAs often regulate development and differenciation as well as cells involved in immune processes.

The biological function of the over 100,000 additional loci that are reported as transcribed by RNA-Sequence data is even less clear. Since the majority are located in introns of known genes and transcribed in the same sense, we interpret these loci as potential byproducts of incomplete splicing, although the distinct patterns formed by these transcript may nonetheless signify something. However, more than 40,000 transcripts are located outside of known features, and their distribution throughout the genome would suggest that the majority are independently transcribed, rather than being run-off products of e.g. protein coding loci. While their expression patterns retain some weak component of tissue-specificity, these loci are often more lowly expressed and can vary between samples, suggesting an involvement in cell state regulation. In order to further characterize the biological roles and relevance of these transcripts, comprehensive and systematic knock-down experiments are required, a low-throughput template of which we demonstrated using a murine podocyte model. The podocyte experiments show that two novel non-coding transcripts, one lincRNA and one antisense, selected because of their proximity to a gene, *Baiap2*, important for formation of podocyte filapodia, appear to regulate this gene and change podocyte morphology and motility in cell culture. This hints at the regulatory potential and the need to characterize the function of the thousands of novel non-coding transcript described in this paper.

In conclusion, we expect that the data set we generated, analyzed, and presented here will serve as an invaluable genomic tool to unravel the biology which underscores many diseases; not just of dogs, but for their masters as well.

The data is freely available from http://genome.ucsc.edu/cgi-bin/hgHubConnect “Broad Canine”. For a detailed description of putative functional characterization of the transcripts, see Table S10.

## Methods

### Ethics statement

A tissue panel (brain, heart, kidney, liver, lung, pancreas, ovary, skin, testis) was created from 23 samples donated from four anonymous euthanized dogs following owner consent and ethical approval. Ethical approval was granted by Uppsala Djurförsöksetiska Nämnd (Permit Number: C 2/12).

### WGA segment finishing

Finishing targets not represented by BACs were addressed by directly finishing whole genome assembly (WGA) segments. The target sequence with an additional ∼40 kb on either side was extracted from canFam2.0, viewed in the genome assembler GAP4 [44], and finished with a combination of fosmid primer walks, PCR and the incorporation of previously unplaced whole genome sequencing (WGS) reads. At sites of SNP haplotype difference, the allele of higher sequence quality was represented. In addition, the longer sequence was represented for indel polymorphisms.

### Automated primer walks of small gaps

After target gaps were identified in the assembly, we identified those that were covered by at least two fosmid templates. Gaps that met this coverage requirement were analyzed with primer3 [45], [46] to find suitable paired primers. Finishing reads (see below) were then generated using primer walks and integrated into a “chunked” sub-assembly. The sub-assembly “chunk” is an approximately 2 kb blunt-end extract of the whole genome assembly surrounding each gap. This integration was done using the shotgun assembly module of GAP4. After all finishing reads were integrated into the chunk assembly, analysis using a novel data structure called a “read coverage signature” (RCS) was completed to determine gap closure or extension and to ensure that the chunk had not been misassembled (see explanation below). The consensus sequence from the chunks that satisfied the RCS analysis was subsequently patched into the whole genome assembly at the positions defined when the chunk was created.

### Read coverage signature

A read coverage signature (RCS) is a data structure that serves as a landmark for cross-assembly navigation. It is constructed by iterating over each read that covers a consensus base in the assembly and hashing the read name, the position in the read, and the base call in the read. A catalog of RCSs can be computed for an entire assembly or for subsections of an assembly. After these catalogs are computed, an area of interest in one assembly can be found in another assembly which shares the same reads by comparing the RCSs.

### Generation of finishing reads from autowalks

Automated primer reads were generated with BigDye and GB (1∶4 dGTP:BigDye, Life Technologies) sequencing chemistries. Even though the dog genome has GC content similar to the human genome [5], hard GC stops (i.e. regions high in GC cannot be accurately sequenced) are relatively frequent. Several sequencing chemistries and additives were used to address this issue. DMSO (Dimethyl sulfoxide) was added to our GB chemistry with favorable results. For sequences with 70%–80% GC content, we observed a 14-point increase in Phred quality score with the use of 5% DMSO. There was a 7-point increase in Phred quality scores for sequences with 80%–95% GC content as well, and an approximately 50 base increase in Q20, Q30, and Q40 read lengths.

We also observed a marginal improvement over the DMSO/GB mix through the use of Resolver (General Electric) template generation. Some GC stops were recalcitrant to these approaches. 57 clones were submitted as comparative grade finished to avoid extreme efforts to resolve small stretches of GC rich sequence. Most gaps in these clones were closed.

### Work region integration

Regions where finishing targets (BACs and WGA segments) overlapped were designated as work regions, which could then be considered as single units of sequence for patching into the canFam3.1 assembly. The constituent clones and WGA segments were integrated using artificial reads generated from the finished consensus. Rules similar to those employed for finishing WGA segments were used to address haplotype variation. Completed multi-BAC assemblies were patched into the original canFam2.0 whole genome assembly, replacing the pre-existing sequence.

### Integration of improved sequence

Larger regions of interest that underwent manual finishing, such as BACs and work regions (see above), are treated similar to the autowalk “chunks” (see previous) in terms of patching. Their coordinates from a golden path (AGP) defined a whole genome “chunk”, which became a finishing project. When the chunk is finished, its consensus sequence is patched into the whole genome assembly at the positions defined when the chunk was created.

### RNA sequencing (RNA-Seq)

In total, 22 sequencing libraries were generated from commercially sought RNA (blood, brain, heart, kidney, liver, lung, ovary, skeletal muscle, skin, and testis (Zyagen)). Each tissue type was used to generate both a strand-specific poly-A selection library (n = 10) and a duplex-specific nuclease library (DSN; n = 10). Two additional poly-A biological libraries were generated (kidney and brain) to act as biological replicates and to test the robustness of tissue specific transcript predictions. The poly-A selection protocol was used to cover sequences transcribed by polymerase II, whilst the DSN non-selective normalization technique was used to reduce highly abundant transcripts. The latter was so that we could sequence all samples comprehensively, but at lower coverage per locus. All 22 libraries were sequenced on the Illumina platform, using paired-end reads with an insert size of ∼300-600 nucleotides and a strand-specific protocol (dUTP).

### Cross-species comparison pipeline

We mapped annotations from the human genome (Gencode version 9) [47] onto the new genome build using syntenic relationships between the two genomes in a two-step pipeline (Zamani et al. submitted). First, we used the synteny aligner Satsuma [26] to establish syntenic relationships between the human and dog genome sequences. This information was then used to produce a rough mapping of annotated features from the query species to the target species within the syntenic regions. Candidate mappings were subsequently re-aligned using a local alignment strategy between feature boundaries. In a final step, we checked the intron-exon boundaries annotated in this manner for neighboring canonical splice sites and adjusted accordingly if such features were found within 5 nucleotides from the predicted boundaries. While all sequences mapped to canFam2.0 could also be located in canFam3.1, we note that there were 75 transcripts that could be mapped from human onto canFam3.1 due to the recovered sequences, but absent in the canFam2.0 build. Out of the ∼1,000 additional exons in canFam3.1, about 60% are first exons, regions that are known to be rich in GC content and were therefore absent from canFam2.0. Of the ∼18,300 loci that could not be mapped from human due to a lack of orthology, the most prominent group consists of retrotransposed pseudogenes (5,827), followed by uncharacterized novel genes with unconfirmed transcriptional support level (4,134). Together these groups account for more than half of the unmapped gene loci. Gene families with missing members include olfactory receptors (330), immunoglobulin and immunoglobulin -related genes (306), zinc finger proteins (204), microRNAs (209), uncharacterized gene families (143), and keratins and keratin-associated proteins (103).

### RNA-Seq pipeline

We aligned all RNA-Seq reads against the unmasked, euchromatic portion of the dog reference genome (canFam3.1, obtained from EnsEMBL release 68) using the splice-junction mapper Tophat (version 2.0.5) [23] with default parameters. We filtered the resulting read alignments using a quality cut-off of 15 and assembled them into transcript models individually per tissue using the cufflinks package (version 2.0.2) [24]. We performed both steps without a reference annotation to avoid the introduction of biases. We then merged the transcriptome annotations from all tissues using the cuffmerge tool - as part of the cufflinks package - into one consensus annotation for each library preparation and then into one combined annotation across all samples (poly-A and DSN).

### Expanded feature map for canFam3.1

In order to produce a ‘master’ annotation for the new genome build, we combined information from the existing annotation with transcript predictions based on RNA-Seq data (see above). To this end, we merged the unguided cufflinks consensus models (see above) with the existing reference annotation (EnsEMBL release 68) using cuffmerge, as discussed above. The EnsEMBL annotation served as a reference (option -g) to allow us a better classification of transcript models obtained from RNA-Seq compared to established gene models and to identify novel isoforms and loci previously unknown to be expressed. Novel loci that nevertheless had a correspondence in human based on synteny (see above) were marked as such, allowing us to effectively differentiate between reference annotations, novel annotations with human orthologs as well as genuinely novel dog annotations. In order to make the annotation compact, we removed 55,149 intronic sense transcripts that are likely by-products of immature splicing, retaining a total of 194,740 transcripts and 106,079 expressed loci. The data is available as a color-coded BED file, which also contains expression values for each tissue and transcript.

### Comparing transcripts and loci

A locus is defined as the set of transcripts that overlap in at least one exon and are transcribed in the same sense. The same criteria are used when comparing RNA-Seq transcripts and known annotations (Table 2).

### Identification of coding potential

Putative coding capacity of novel transcripts was determined in three complementary ways. First, we identified open reading frames of 50 amino acids or longer. We next used the TranscriptDecoder package (http://sourceforge.net/p/transdecoder) - a collection of scripts that identify open-read frames in nucleotide sequences and compare them to the Pfam database of protein families, release 26 [48]. This rather stringent approach was complemented by a search of translated nucleotide sequences against a set of vertebrate proteins from RefSeq (downloaded in September 2012) using the blastx algorithm from the Blast package (searching only the sense strand, -S 1) [49]. Only Blast hits with a bitscore of over 60 were included into our subsequent analyses in order to remove cases where support for a possible homology in our opinion was too weak. In total, we identified 3,455 hitherto unannotated loci that fall into one of the categories above.

### Expression atlas for canFam3.1

We determined a normalized expression estimate for all loci in our expanded feature map and across our 19 (21 including replicates for brain and kidney) samples using the cuffdiff tool from the cufflinks package [24], correcting for sequencing biases (-b) and multi-mapped reads (-u) and employing a upper quartile normalization (-N).

### Experimental verification of novel transcript expression

A tissue panel (brain, heart, kidney, liver, lung, pancreas, ovary, skin, testis) was created from 23 samples donated from four anonymous euthanized dogs. Total RNA was extracted from ∼50 milligrams of tissue using the RNeasy mini kit (Qiagen) following the manufacturers instruction and with an additional Turbo DNA-free treatment (Ambion) to remove genomic DNA. RNA was quantified on a Bioanalyser (RIN between 7 and 9 for all samples) prior to cDNA synthesis using one microgram of total RNA and the Advantage RT-for-PCR kit (Clontech). No gDNA contamination was identified and so the expression of 22 novel loci were measured and normalised against *ACTB* (primers, Table S7). Loci were defined according to physical location, either intergenic or antisense, and tissue specificity, where tissue specific loci were required to be expressed at an FPKM>5.0 in a single tissue and <2.0 in all others.

### Cross species validation of novel transcripts

We identified a set of novel multi-exonic canine transcripts that overlapped known genes in the antisense direction. Intron spanning primers were designed to amplify products from dogs and from orthologous human locations (Table S9). Intron spanning primers for two proposed lincRNAs from the canine transcriptome were designed to amplify products from canine, mouse, and human kidney RNA (Table S9). To perform standard RT-PCR, 2 ug each of canine kidney RNA (Zyagen, San Diego, CA), mouse and human kidney RNA (Ambion, Austin, TX) were treated with Turbo DNAse (Invitrogen) with subsequent cDNA synthesis using Superscript II RT (Invitrogen). Specific PCR products were then confirmed by Sanger sequencing.

### Functional characterization of novel transcripts in murine podocytes

To design shRNA constructs, multiple 21-mer targets were identified for the murine *BAIAP2* coding, *BAIAP2* lincRNA, and *BAIAP2* antisense transcripts using the siRNA Selection Program (http://jura.wi.mit.edu/bioc/siRNAext/); coding: GCAGCTGGCCTAGAACGTAAT, CTATTTCGATGCTCTGGTAAA; lincRNA: GTAATTGACAAGTCCTTATTT, ACCATACCATGCTGGTCTAAA, CACAGCTCCTTGTTGGCTTTC; antisense: CCCAGTGACTTCAGACAACAT, GACTCAGCCAATCACGGTTAA, AGGGAAGTTCTGAGATCCATC, CCAAGCCTCATGAACTCATTC.

Oligos were generated with the following sequence CCGG-21 bp sense-CTCGAG-21 bp antisense-TTTTTG and subcloned into the pLKO.1 plasmid (Addgene, Cambridge, MA). The standard pLKO.1 scrambled plasmid was used as a control. All plasmids were Sanger sequenced to verify correct subcloning. To produce shRNA lentivirus, HEK 293-T cells grown in standard growth media (DMEM, 10% FBS+pen/strep) were transfected with each shRNA-pLKO.1 plasmid, along with the packaging plasmid (delta R8.91, Addgene) and the envelope plasmid (VSVG, Addgene) in a 10∶10∶1 ratio, respectively using Mirus LT (Madison, WI) transfection reagent ((3∶1 ratio plasmid DNA (ug) to Mirus reagent (uL)). Virus containing media was collected after 72 hours, filtered, and frozen immediately in aliquots at −80 degrees. Conditionally immortalized murine podocytes [50] were propagated under permissive conditions and seeded (5×10^5^) in 6 well plates. Upon reaching 90% confluence, the cells were differentiated into podocytes (characterized by positive WT-1) upon shifting to 37 degrees in the absence of gamma interferon. The differentiated podocytes were infected with experiment specific (scrambled, coding, lincRNA, anti sense) lentivirus six days after thermoshift. For morphological analysis, podocytes were seeded on coverslips and stained with a phalloidin actin stain (Invitrogen, Carlsbad, CA) 4 days after infection. To perform the migration assay, a single wound was performed in the center of each well with a 200-ul pipette tip four days after infection. Following three hours of serum starvation to synchronize the podocytes, the cells were washed three times with PBS to remove debris. Using a grid as a marker, images to assess cell migration were obtained at 0, 24, 48 hours using a×10 phase contrast objective and the cells were fixed and stained with DAPI (Invitrogen) at 48 hours. The number of cells that migrated into the scratch (determined at T = 0) at 48 hours were recorded for each experiment. The data for each condition represent the mean +/− SEM of six experiments. For quantitative PCR, RNA was isolated from podocytes by RNeasy mini kit (Qiagen) and cDNA synthesis was carried out via Superscript II RT (Invitrogen) using standard conditions. Quantitative assays of BAIAP2 expression (FP: cggctcacggaaaatgtcta, RP: gcgtctcttccaactggttc) were carried out on a Light Cycler 480 (Roche) using Sybr Green Master Mix (Roche) and HPRT (FP: ctggtgaaaaggacctctcgaa, RP: ctgaagtactcattatagtcaagggcat) as the internal control. Websites GAP4: http://staden.sourceforge.net/manual/gap4_unix_2.html.

Primer3: http://bioinfo.ut.ee/primer3-0.4.0/primer3/.

### Data access

The RNA Seq data is available in the short read archive under NCBI BioProject PRJNA7882 and accessions SRX111061 - SRX111071, SRX146606- SRX146608.

## Supporting Information

Figure S1 **Expression profiles of annotation categories.**
(DOCX)

Figure S2 **qPCR assessment of BAIAP2 expression in mouse podocytes treated with shRNAs.**
(DOCX)

Table S1 **Read count and mean insert size for the 22 RNA-seq libraries.**
(DOCX)

Table S2 A. Locus-level comparision of polyA-selected, tissue-specific transcript models. B. Locus-level comparison of DSN-selected, tissue-specific transcript models.
(DOCX)

Table S3 **Genes mapped from human to canFam3.1.**
(XLS)

Table S4 **Functional clustering of antisense host genes.**
(DOCX)

Table S5 **Canonical pathways with multiple genes containing antisense transcripts.**
(XLSX)

Table S6 **Canonical pathways with multiple genes containing lincRNA transcripts.**
(XLS)

Table S7 **Novel non-coding transcripts validated by Q-PCR.** Pink: tissues not matching in discovery and validation set; green: Strong expression in discovery dataset + validation; light green: Weak expression in discover data set + validation; grey: expression detected in validation dataset.
(XLSX)

Table S8 **Primers for expression panel in multiple canine tissues.**
(XLSX)

Table S9 **Primer sets used for validation of novel canine antisense and intergenic transcripts in human and mouse kidney RNA.**
(DOCX)

Table S10 **Transcripts by category and putative function.**
(DOCX)
