# Supplementary material for: An Improved Canine Genome and a Comprehensive Catalogue of Coding Genes and Non-Coding Transcripts
Source: PLoS One. 2014 Mar 13;9(3):e91172. doi: 10.1371/journal.pone.0091172 (PMC3953330; doi:10.1371/journal.pone.0091172)
Supplement: Figure S1 — Expression profiles of annotation categories. (DOCX) [file pone.0091172.s001.docx]

**Figure S1. Expression profiles of annotation categories.** A comparision of expression strength across libraries and annotation categories (known genes, novel intergenic and novel antisense loci) shows a more narrow breadth of expression strengths for novel loci, with the majority of annotations being restricted to very low expression levels. Interestingly, the top percentile of novel annotations in selected DSN libraries exhibit FPKM values in line with or greater than known protein coding genes.
