# Supplementary material for: An Improved Canine Genome and a Comprehensive Catalogue of Coding Genes and Non-Coding Transcripts
Source: PLoS One. 2014 Mar 13;9(3):e91172. doi: 10.1371/journal.pone.0091172 (PMC3953330; doi:10.1371/journal.pone.0091172)
Supplement: Figure S2 — qPCR assessment of BAIAP2 expression in mouse podocytes treated with shRNAs. (DOCX) [file pone.0091172.s002.docx]

**Figure S2. qPCR assessment of BAIAP2 expression in mouse podocytes treated with shRNAs.** Treatment of podocytes with shRNAs to the BAIAP2 coding region and lincRNA resulted in decreased BAIAP2 expression, while treatment with shRNAs to the antisense transcript did not alter levels of the BAIAP2 transcript compared to scrambled control. All samples were normalized to HPRT expression as the internal control.
