# Supplementary material for: An Improved Canine Genome and a Comprehensive Catalogue of Coding Genes and Non-Coding Transcripts
Source: PLoS One. 2014 Mar 13;9(3):e91172. doi: 10.1371/journal.pone.0091172 (PMC3953330; doi:10.1371/journal.pone.0091172)
Supplement: Table S1 — Read count and mean insert size for the 22 RNA-seq libraries. (DOCX) [file pone.0091172.s003.docx]

**Table S1. Read count and mean insert size for the 22 RNA-seq libraries**

|  | **Poly-A** |  | **DSN** |  |
| --- | --- | --- | --- | --- |
|  | Reads (aligned) | Insert size  (bp, mean) | Reads (aligned) | Insert size  (bp, mean) |
| **Blood** | 32433860 | 390.0 | 20831578 | 337.7 |
| **Brain** | 35663099 | 460.0 | 28305687 | 331.3 |
| **Heart** | 38687230 | 401.1 | 29553230 | 292.5 |
| **Kidney** | 44620745 | 404.4 | 25950750 | 323.7 |
| **Liver** | 38917942 | 654.7 | 46666197 | 256.3 |
| **Lung** | 34381493 | 444.1 | 29452507 | 340.4 |
| **Muscle** | 36654453 | 445.4 | 23760815 | 394.1 |
| **Ovary** | 38856765 | 413.7 | 37213454 | 290.7 |
| **Skin** | 28502112 | 459.5 | 5486454* | 230.1 |
| **Testis** | 40506034 | 408.7 | 37497376 | 239.4 |
|  |  |  |  |  |
| **Brain replicate** | 99109084 | 489.7 |  |  |
| **Kidney replicate** | 105332473 | 594.7 |  |  |

* Excluded from subsequent analyses due to poor alignment performance
