# Supplementary material for: An Improved Canine Genome and a Comprehensive Catalogue of Coding Genes and Non-Coding Transcripts
Source: PLoS One. 2014 Mar 13;9(3):e91172. doi: 10.1371/journal.pone.0091172 (PMC3953330; doi:10.1371/journal.pone.0091172)
Supplement: Table S2 — A. Locus-level comparision of polyA-selected, tissue-specific transcript models. B. Locus-level comparison of DSN-selected, tissue-specific transcript models. (DOCX) [file pone.0091172.s004.docx]

**Table S2-A. Locus-level comparision of polyA-selected, tissue-specific transcript models**

|  | **blood** | **brain** | **heart** | **kidney** | **liver** | **lung** | **ovary** | **muscle** | **skin** | **Testis** |
| --- | --- | --- | --- | --- | --- | --- | --- | --- | --- | --- |
| **blood** | **30325** | 21845 | 19334 | 21299 | 18809 | 22957 | 23686 | 18117 | 23823 | 20927 |
| **brain** | 19575 | **33486** | 20629 | 22671 | 18824 | 23841 | 26196 | 19039 | 24963 | 23216 |
| **heart** | 17297 | 20672 | **23807** | 20031 | 17402 | 20521 | 21310 | 18766 | 21107 | 19228 |
| **kidney** | 18519 | 22079 | 19539 | **28420** | 18637 | 22339 | 23679 | 18142 | 23078 | 20789 |
| **liver** | 18876 | 20709 | 19379 | 21524 | **25431** | 21244 | 21859 | 18206 | 21570 | 19822 |
| **lung** | 19680 | 22884 | 19504 | 21866 | 18171 | **29493** | 24980 | 17925 | 24352 | 21367 |
| **ovary** | 22496 | 27934 | 22693 | 25804 | 20718 | 27898 | **39976** | 20633 | 30274 | 25911 |
| **muscle** | 16891 | 19693 | 19653 | 19333 | 17127 | 19558 | 20240 | **22221** | 20454 | 18440 |
| **skin** | 19293 | 23078 | 19489 | 21739 | 17892 | 23252 | 25641 | 18010 | **34335** | 21831 |
| **testis** | 18278 | 23026 | 19444 | 21261 | 17788 | 22058 | 23866 | 17932 | 23278 | **41070** |

**Table S2-B. Locus-level comparison of DSN-selected, tissue-specific transcript models**

|  | **blood** | **brain** | **heart** | **kidney** | **liver** | **lung** | **muscle** | **ovary** | **testes** |
| --- | --- | --- | --- | --- | --- | --- | --- | --- | --- |
| **blood** | **69030** | 41322 | 23459 | 14285 | 28958 | 53358 | 36180 | 21206 | 10999 |
| **brain** | 25528 | **64657** | 19746 | 13030 | 21922 | 36685 | 26053 | 19279 | 10849 |
| **heart** | 23313 | 30344 | **43659** | 11767 | 20151 | 34744 | 30795 | 16073 | 8713 |
| **kidney** | 19887 | 24614 | 16154 | **38059** | 17105 | 28035 | 22557 | 11232 | 6338 |
| **liver** | 50037 | 58189 | 35152 | 18511 | **96231** | 70346 | 53842 | 22217 | 11239 |
| **lung** | 36807 | 39004 | 23374 | 15241 | 27764 | **67842** | 32253 | 23015 | 12208 |
| **muscle** | 17838 | 21424 | 16971 | 11018 | 17368 | 24976 | **31006** | 14674 | 9031 |
| **ovary** | 39787 | 50893 | 29931 | 13980 | 27236 | 58913 | 44642 | **83665** | 8596 |
| **testes** | 16096 | 19874 | 12553 | 7062 | 12105 | 22218 | 17806 | 8252 | **33857** |

* Skin was excluded from this analysis based on poor alignment performance
