# Supplementary material for: An Improved Canine Genome and a Comprehensive Catalogue of Coding Genes and Non-Coding Transcripts
Source: PLoS One. 2014 Mar 13;9(3):e91172. doi: 10.1371/journal.pone.0091172 (PMC3953330; doi:10.1371/journal.pone.0091172)
Supplement: Table S4 — Functional clustering of antisense host genes. (DOCX) [file pone.0091172.s006.docx]

**Table S4. Functional clustering of antisense host genes**

| ID | Associated Network Functions | Score |
| --- | --- | --- |
| 1 | DNA Replication, Recombination, and Repair, Energy Production, Nucleic Acid Metabolism | 39 |
| 2 | Post-Translational Modification, Hereditary Disorder, Neurological Disease | 37 |
| 3 | Hereditary Disorder, Ophthalmic Disease, Nervous System Development and Function | 37 |
| 4 | Nervous System Development and Function, Cell Signaling, Cell-To-Cell Signaling and Interaction | 37 |
| 5 | RNA Post-Transcriptional Modification, RNA Damage and Repair, Amino Acid Metabolism | 36 |
