# Supplementary material for: An Improved Canine Genome and a Comprehensive Catalogue of Coding Genes and Non-Coding Transcripts
Source: PLoS One. 2014 Mar 13;9(3):e91172. doi: 10.1371/journal.pone.0091172 (PMC3953330; doi:10.1371/journal.pone.0091172)
Supplement: Table S9 — Primer sets used for validation of novel canine antisense and intergenic transcripts in human and mouse kidney RNA. (DOCX) [file pone.0091172.s011.docx]

**Table S9.** **Primer sets used for validation of novel canine antisense and intergenic transcripts in human and mouse kidney RNA**. Six of the seven novel antisense loci were amplified from homologous human locations using human kidney RNA suggesting conservation of loci across species (amplification of XLOC_022656* was unsuccessful). There were no previously defined antisense transcripts at these loci in humans or dogs. Similarly, two intergenic RNAs identified in dogs were successfully amplified from mouse and human kidney RNA.
