# Supplementary material for: An Improved Canine Genome and a Comprehensive Catalogue of Coding Genes and Non-Coding Transcripts
Source: PLoS One. 2014 Mar 13;9(3):e91172. doi: 10.1371/journal.pone.0091172 (PMC3953330; doi:10.1371/journal.pone.0091172)
Supplement: Table S10 — Transcripts by category and putative function. (DOCX) [file pone.0091172.s012.docx]

**Table S10. Transcripts by category and putative function.**

|  | Category | Transcript counts | Putative function |
| --- | --- | --- | --- |
| 1 | FULL_SENSE.protein_coding_high | 73,357 | **Protein coding** |
| 2 | PARTIAL_SENSE.protein_coding_high | 26,825 | **Protein coding** |
| 3 | FULL_ANTI.antisense_high | 552 | **Antisense** |
| 4 | PARTIAL_ANTI.antisense_high | 4,084 | **Antisense** |
| 5 | INTRONIC_ANTI.antisense_low | 659 | Antisense |
| 6 | SPLICED.intergenic_noncoding_spliced_high | 7,224 | **lincRNA** |
| 7 | SINGLE.intergenic_noncoding_single_high | 36,483 | **Non-coding** |
| 8 | PARTIAL_ANTI.intergenic_noncoding_low | 2,314 | N/A |
| 9 | SPLICED.intergenic_noncoding_low | 1,124 | N/A |
| 10 | SINGLE.intergenic_noncoding_low | 2,792 | N/A |
| 11 | INTRONIC_SENSE.na | 30,984 | **N/A** |
| 12 | FULL_ANTI.intergenic_noncoding_low | 191 | N/A |
| 13 | INTRONIC_ANTI.intergenic_noncoding_low | 1,841 | N/A |
| 14 | INTRONIC_ANTI.na | 1,209 | N/A |
| 15 | FULL_ANTI.na | 823 | **N/A** |
| 16 | PARTIAL_ANTI.na | 446 | **N/A** |
| 17 | SPLICED.NA | 713 | **N/A** |
| 18 | SINGLE.NA | 3,119 | **N/A** |

We divided transcripts into 18 different categories, as listed above. We classified categories 1 and 2 as protein coding based on sequence overlaps between the transcript and protein coding genes annotated by Ensembl in the dog genome, and/or overlaps with human orthologs mapped onto the dog genome (total = 100,182). Full overlaps (1) require that all exons of the RNA-Seq transcripts overlap annotated exons, and vice versa, and on the same strand of transcription. Partial overlaps (2) require at least one overlapping exon on the same strand. For antisense transcripts (total = 4,636) we require overlaps on the opposite strand, and distinguish between full overlaps (3), partial overlaps (4). We also list overlaps in introns (5) with an annotated protein coding dog gene, after running *Cuffmerge*. Long intergenic non-coding RNAs (lincRNA, 6) are required to fall outside of annotated protein coding genes, both dog and human orthologs, but may overlap loci annotated as lincRNA, and have to contain intron-exon structures. Single intergenic RNAs (7) contain single exons only, and fall outside of any annotated locus. Category 8 lists transcripts that overlap human orthologs in antisense direction, but do not overlap loci annotated in dog, while category 9 and 10 list intergenic transcripts that fall outside of annotated loci, but have the potential to be protein coding based on similarity to known proteins or protein domains, either with multiple exons (9) or single exons (10). The intronic sense transcript listed here (11) are sequences kept after running *Cuffmerge*, and reside in introns of protein coding genes in sense direction. Categories 12 through 18 list the remaining cases, antisense transcripts to non-coding annotated genes (12), intronic antisense transcripts to annotated non-coding genes (13), while the remaining cases 14 through 18 could not be unambiguously characterized. The single-exon intergenic transcripts analysed in the main text include categories 7, 15, 16, 17 and 18.
